# GENCODE 2025: reference gene annotation for human and mouse

**DOI:** 10.1093/nar/gkae1078

**Published:** 2024-11-20

**Authors:** Jonathan M Mudge, Sílvia Carbonell-Sala, Mark Diekhans, Jose Gonzalez Martinez, Toby Hunt, Irwin Jungreis, Jane E Loveland, Carme Arnan, If Barnes, Ruth Bennett, Andrew Berry, Alexandra Bignell, Daniel Cerdán-Vélez, Kelly Cochran, Lucas T Cortés, Claire Davidson, Sarah Donaldson, Cagatay Dursun, Reham Fatima, Matthew Hardy, Prajna Hebbar, Zoe Hollis, Benjamin T James, Yunzhe Jiang, Rory Johnson, Gazaldeep Kaur, Mike Kay, Riley J Mangan, Miguel Maquedano, Laura Martínez Gómez, Nourhen Mathlouthi, Ryan Merritt, Pengyu Ni, Emilio Palumbo, Tamara Perteghella, Fernando Pozo, Shriya Raj, Cristina Sisu, Emily Steed, Dulika Sumathipala, Marie-Marthe Suner, Barbara Uszczynska-Ratajczak, Elizabeth Wass, Yucheng T Yang, Dingyao Zhang, Robert D Finn, Mark Gerstein, Roderic Guigó, Tim J P Hubbard, Manolis Kellis, Anshul Kundaje, Benedict Paten, Michael L Tress, Ewan Birney, Fergal J Martin, Adam Frankish

**Affiliations:** European Molecular Biology Laboratory, European Bioinformatics Institute, Wellcome Genome Campus, Hinxton, Cambridge CB10 1SD, UK; Centre for Genomic Regulation (CRG), The Barcelona Institute of Science and Technology, Dr. Aiguader 88, Barcelona 08003 Catalonia, Spain; UC Santa Cruz Genomics Institute, 2300 Delaware Avenue, University of California, Santa Cruz, CA 95060, USA; European Molecular Biology Laboratory, European Bioinformatics Institute, Wellcome Genome Campus, Hinxton, Cambridge CB10 1SD, UK; European Molecular Biology Laboratory, European Bioinformatics Institute, Wellcome Genome Campus, Hinxton, Cambridge CB10 1SD, UK; Computer Science and Artificial Intelligence Lab, Massachusetts Institute of Technology, 32 Vassar St, Cambridge, MA 02139, USA; The Broad Institute of MIT and Harvard, 415 Main Street, Cambridge, MA 02142, USA; European Molecular Biology Laboratory, European Bioinformatics Institute, Wellcome Genome Campus, Hinxton, Cambridge CB10 1SD, UK; Centre for Genomic Regulation (CRG), The Barcelona Institute of Science and Technology, Dr. Aiguader 88, Barcelona 08003 Catalonia, Spain; European Molecular Biology Laboratory, European Bioinformatics Institute, Wellcome Genome Campus, Hinxton, Cambridge CB10 1SD, UK; European Molecular Biology Laboratory, European Bioinformatics Institute, Wellcome Genome Campus, Hinxton, Cambridge CB10 1SD, UK; European Molecular Biology Laboratory, European Bioinformatics Institute, Wellcome Genome Campus, Hinxton, Cambridge CB10 1SD, UK; European Molecular Biology Laboratory, European Bioinformatics Institute, Wellcome Genome Campus, Hinxton, Cambridge CB10 1SD, UK; Bioinformatics Unit, Spanish National Cancer Research Centre (CNIO), Calle Melchor Fernandez Almagro, 3, 28029 Madrid, Spain; Department of Computer Science, Stanford University, 353 Jane Stanford Way, Stanford, CA, USA; European Molecular Biology Laboratory, European Bioinformatics Institute, Wellcome Genome Campus, Hinxton, Cambridge CB10 1SD, UK; European Molecular Biology Laboratory, European Bioinformatics Institute, Wellcome Genome Campus, Hinxton, Cambridge CB10 1SD, UK; European Molecular Biology Laboratory, European Bioinformatics Institute, Wellcome Genome Campus, Hinxton, Cambridge CB10 1SD, UK; Program in Computational Biology and Bioinformatics, Yale University, New Haven, CT 06520, USA; Department of Molecular Biophysics and Biochemistry, Yale University, New Haven, CT 06520, USA; European Molecular Biology Laboratory, European Bioinformatics Institute, Wellcome Genome Campus, Hinxton, Cambridge CB10 1SD, UK; European Molecular Biology Laboratory, European Bioinformatics Institute, Wellcome Genome Campus, Hinxton, Cambridge CB10 1SD, UK; UC Santa Cruz Genomics Institute, 2300 Delaware Avenue, University of California, Santa Cruz, CA 95060, USA; European Molecular Biology Laboratory, European Bioinformatics Institute, Wellcome Genome Campus, Hinxton, Cambridge CB10 1SD, UK; Computer Science and Artificial Intelligence Lab, Massachusetts Institute of Technology, 32 Vassar St, Cambridge, MA 02139, USA; The Broad Institute of MIT and Harvard, 415 Main Street, Cambridge, MA 02142, USA; Program in Computational Biology and Bioinformatics, Yale University, New Haven, CT 06520, USA; Department of Molecular Biophysics and Biochemistry, Yale University, New Haven, CT 06520, USA; Department of Medical Oncology, Bern University Hospital, Murtenstrasse 35, 3008 Bern, Switzerland; School of Biology and Environmental Science, University College Dublin,, Belfield, Dublin 4 D04 V1W8, Ireland; Centre for Genomic Regulation (CRG), The Barcelona Institute of Science and Technology, Dr. Aiguader 88, Barcelona 08003 Catalonia, Spain; European Molecular Biology Laboratory, European Bioinformatics Institute, Wellcome Genome Campus, Hinxton, Cambridge CB10 1SD, UK; Computer Science and Artificial Intelligence Lab, Massachusetts Institute of Technology, 32 Vassar St, Cambridge, MA 02139, USA; The Broad Institute of MIT and Harvard, 415 Main Street, Cambridge, MA 02142, USA; Genetics Training Program, Harvard Medical School, Boston, MA 02115, USA; Bioinformatics Unit, Spanish National Cancer Research Centre (CNIO), Calle Melchor Fernandez Almagro, 3, 28029 Madrid, Spain; Bioinformatics Unit, Spanish National Cancer Research Centre (CNIO), Calle Melchor Fernandez Almagro, 3, 28029 Madrid, Spain; European Molecular Biology Laboratory, European Bioinformatics Institute, Wellcome Genome Campus, Hinxton, Cambridge CB10 1SD, UK; European Molecular Biology Laboratory, European Bioinformatics Institute, Wellcome Genome Campus, Hinxton, Cambridge CB10 1SD, UK; Program in Computational Biology and Bioinformatics, Yale University, New Haven, CT 06520, USA; Department of Molecular Biophysics and Biochemistry, Yale University, New Haven, CT 06520, USA; Centre for Genomic Regulation (CRG), The Barcelona Institute of Science and Technology, Dr. Aiguader 88, Barcelona 08003 Catalonia, Spain; Centre for Genomic Regulation (CRG), The Barcelona Institute of Science and Technology, Dr. Aiguader 88, Barcelona 08003 Catalonia, Spain; Departament de Ciències Experimentals i de la Salut, Universitat Pompeu Fabra (UPF), Carrer de la Mercè, 12, Ciutat Vella 08002 Barcelona, Spain; Bioinformatics Unit, Spanish National Cancer Research Centre (CNIO), Calle Melchor Fernandez Almagro, 3, 28029 Madrid, Spain; European Molecular Biology Laboratory, European Bioinformatics Institute, Wellcome Genome Campus, Hinxton, Cambridge CB10 1SD, UK; Department of Molecular Biophysics and Biochemistry, Yale University, New Haven, CT 06520, USA; Department of Life Sciences, Brunel University London, Kingston Lane, Uxbridge, London UB8 3PH, UK; European Molecular Biology Laboratory, European Bioinformatics Institute, Wellcome Genome Campus, Hinxton, Cambridge CB10 1SD, UK; European Molecular Biology Laboratory, European Bioinformatics Institute, Wellcome Genome Campus, Hinxton, Cambridge CB10 1SD, UK; European Molecular Biology Laboratory, European Bioinformatics Institute, Wellcome Genome Campus, Hinxton, Cambridge CB10 1SD, UK; Department of Computational Biology of Noncoding RNA, Institute of Bioorganic Chemistry, Polish Academy of Sciences, Noskowskiego12/14, 61-704 Poznan, Poland; European Molecular Biology Laboratory, European Bioinformatics Institute, Wellcome Genome Campus, Hinxton, Cambridge CB10 1SD, UK; Department of Molecular Biophysics and Biochemistry, Yale University, New Haven, CT 06520, USA; Institute of Science and Technology for Brain-Inspired Intelligence, Fudan University, 220 Handan Road, Shanghai 200433, China; Program in Computational Biology and Bioinformatics, Yale University, New Haven, CT 06520, USA; Department of Molecular Biophysics and Biochemistry, Yale University, New Haven, CT 06520, USA; European Molecular Biology Laboratory, European Bioinformatics Institute, Wellcome Genome Campus, Hinxton, Cambridge CB10 1SD, UK; Program in Computational Biology and Bioinformatics, Yale University, New Haven, CT 06520, USA; Department of Molecular Biophysics and Biochemistry, Yale University, New Haven, CT 06520, USA; Centre for Genomic Regulation (CRG), The Barcelona Institute of Science and Technology, Dr. Aiguader 88, Barcelona 08003 Catalonia, Spain; Departament de Ciències Experimentals i de la Salut, Universitat Pompeu Fabra (UPF), Carrer de la Mercè, 12, Ciutat Vella 08002 Barcelona, Spain; Department of Medical and Molecular Genetics, King’s College London, Guys Hospital, Great Maze Pond, London SE1 9RT, UK; ELIXIR Hub, Wellcome Genome Campus, Hinxton, Cambridge CB10 1SD, UK; Computer Science and Artificial Intelligence Lab, Massachusetts Institute of Technology, 32 Vassar St, Cambridge, MA 02139, USA; The Broad Institute of MIT and Harvard, 415 Main Street, Cambridge, MA 02142, USA; Department of Computer Science, Stanford University, 353 Jane Stanford Way, Stanford, CA, USA; Department of Genetics, Stanford University, Stanford, CA, USA; UC Santa Cruz Genomics Institute, 2300 Delaware Avenue, University of California, Santa Cruz, CA 95060, USA; Bioinformatics Unit, Spanish National Cancer Research Centre (CNIO), Calle Melchor Fernandez Almagro, 3, 28029 Madrid, Spain; European Molecular Biology Laboratory, European Bioinformatics Institute, Wellcome Genome Campus, Hinxton, Cambridge CB10 1SD, UK; European Molecular Biology Laboratory, European Bioinformatics Institute, Wellcome Genome Campus, Hinxton, Cambridge CB10 1SD, UK; European Molecular Biology Laboratory, European Bioinformatics Institute, Wellcome Genome Campus, Hinxton, Cambridge CB10 1SD, UK

## Abstract

GENCODE produces comprehensive reference gene annotation for human and mouse. Entering its twentieth year, the project remains highly active as new technologies and methodologies allow us to catalog the genome at ever-increasing granularity. In particular, long-read transcriptome sequencing enables us to identify large numbers of missing transcripts and to substantially improve existing models, and our long non-coding RNA catalogs have undergone a dramatic expansion and reconfiguration as a result. Meanwhile, we are incorporating data from state-of-the-art proteomics and Ribo-seq experiments to fine-tune our annotation of translated sequences, while further insights into function can be gained from multi-genome alignments that grow richer as more species’ genomes are sequenced. Such methodologies are combined into a fully integrated annotation workflow. However, the increasing complexity of our resources can present usability challenges, and we are resolving these with the creation of filtered genesets such as MANE Select and GENCODE Primary. The next challenge is to propagate annotations throughout multiple human and mouse genomes, as we enter the pangenome era. Our resources are freely available at our web portal www.gencodegenes.org, and via the Ensembl and UCSC genome browsers.

## Graphical Abstract

**Graphical Abstract:**
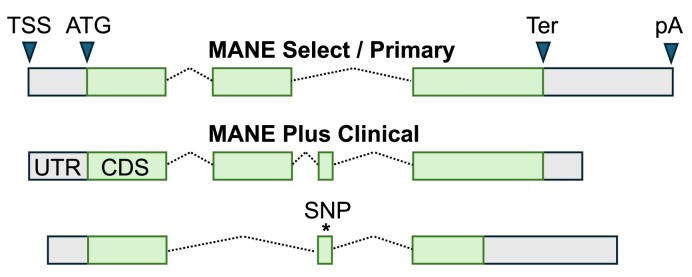

## Introduction to GENCODE

Ensembl-GENCODE (henceforth GENCODE) produced its first gene annotations in 2005 as part of the pilot phase of the nascent human ENCODE project (1), building on the initial annotation efforts of the Human Genome Project (2). Having established the feasibility of using a largely manual approach to gene annotation (1,3), the first full human and mouse GENCODE ‘genebuilds’ were released to the public in 2009 and 2011, respectively. Today, following substantial progress across all branches of ‘omics, annotation is a significantly more advanced process compared with the early days. The situation in transcriptomics is especially striking; a single run on a PacBio or Oxford Nanopore (ONT) flow cell can now generate more complementary DNAs (cDNAs) than were present in the whole of the INSDC Human sequence databases in 2009 (4). Meanwhile, our project now has access to technologies that did not exist 20 years ago, such as Ribo-seq, which in effect sequences RNA undergoing translation (5). However, although such advances allow us to probe deeper into the functional secrets of the genome, the sheer scale of data availability produces significant methodological hurdles for our project, especially given our historical deployment of expert human annotation to produce ‘reference-quality’ models. Our challenge then is how to capture this staggering complexity in our genebuilds, and also how to help our users plot their pathway through this.

Our central goals have remained essentially unchanged since the beginning. We aim to annotate all human and mouse protein-coding genes, long non-coding RNAs (lncRNAs), pseudogenes and small RNAs (these are annotated by a computational pipeline linked to external databases and are not discussed further). In practice, the majority of genes produce a series of distinct transcripts, which differ primarily in terms of their exon-intron structures due to alternative splicing. Thus, our process is better understood in many respects as *transcript* annotation, and the ‘biotype’ of the gene, i.e. the functional classification we set, is defined by the biotype of the transcripts it contains. A protein-coding gene, for example, includes at least one protein-coding transcript, and the accurate annotation of coding sequences (CDSs) remains a core drive of our project given the special importance of these regions in genomic and clinical science. GENCODE annotations contain rich information beyond these basic gene and transcript ‘biotypes’. In particular, we use an ‘attribute’-tagging system to highlight specific functional insights that can be made for given models, such as CDS that are subject to particular phenomena such as stop codon readthrough or translational initiation via non-ATG codons. A full list of these tags, as well as comprehensive descriptions for each gene or transcript ‘biotype’, can be found on the GENCODE website (gencodegenes.org).

### An overview of progress in GENCODE

Table 1 summarizes the annotation statistics from the most recent GENCODE genebuild releases for human and mouse, compared against equivalent data from ∼2 years ago as presented in our previous report by Frankish *et al.* (6). The major trend over this time for both species is the substantial addition of new lncRNA genes and transcripts, while changes in protein-coding gene and transcript counts are more incremental. We discuss both of these aspects in detail below. However, we note that these values report net changes, and emphasize the fact that annotation is not simply a case of adding new models. Thus, over 37 000 existing models have been modified for human and mouse combined in the last two years. This process may involve the switching of biotypes where this is deemed appropriate, e.g. a non-coding model may be changed to coding or vice versa. Also, exon structures can be adjusted. In particular, models are often not ‘full-length’ with respect to the cellular transcript they represent, and the completion of ‘partial’ models is currently a major drive of the project. Indeed, the extension of a given model often changes its biotype, for example, when the full-length CDS can then be identified. Overall, close to a quarter of human and mouse protein-coding genes, lncRNA genes or pseudogenes that existed in GENCODE annotations two years ago have been modified in some way over the intervening period.

**Table 1.** Total numbers of genes and transcripts in the GENCODE 47 (human) and GENCODE M36 (mouse) releases (October 2024), compared against previous releases v41 and M30 (April 2022). Counts are separated into gene and transcript functional biotypes. Readthrough loci that span multiple individual protein-coding genes are excluded from these counts. For pseudogenes and soluble RNAs (sRNAs), a gene by definition contains a single transcript model

			Protein coding	lncRNA	Pseudogene	sRNA	IG/TR
Human	GENCODE 47	**Genes**	**19 433**	**35 934**	**14 703**	**7565**	**411**
		Transcripts	170 270	191 106	/	/	/
	GECODE 41	**Genes**	**19 370**	**19 095**	**14 737**	**7566**	**410**
		Transcripts	167 599	54 291	/	/	/
Mouse	GENCODE M36	**Genes**	**21 470**	**36 172**	**13 769**	**6105**	**493**
		Transcripts	101 225	156 135	/	/	/
	GENCODE M30	**Genes**	**21 668**	**14 525**	**13 468**	**6105**	**494**
		Transcripts	101 716	25 419	/	/	/

GENCODE initially approached human and mouse gene annotation using a ‘chromosome by chromosome’ workflow. In effect, expert manual annotators moved along each chromosome 5′ to 3′, one locus at a time, building transcript models as required. Once the ‘first pass’ manual annotations of these genomes were completed several years ago, our process became more modular and focused on specific sub-projects. These may consider the whole genome, for example, incorporating new transcriptomics datasets, or alternatively identify particular genomic regions or gene classes for improvement. Here, we will outline progress in GENCODE in recent months according to the deployment of such workflows. However, we emphasize that a major strength of our project is that such efforts are not individualized; rather, each can be seen as an integrated part of our wider annotation drive.

### Annotating transcript models

The major focus in GENCODE human and mouse transcript annotation over the last five years has been developing and deploying the Capture Long-read Sequencing (CLS) pipeline (7). We recently completed the third phase of this project—CLS3—to expand and improve lncRNA annotations in both species. Briefly, this project started with the design of capture arrays, targeting over 300 000 regions of the human and mouse genomes for which a deeper appraisal of their transcriptional potential was considered promising. PacBio and Oxford Nanopore (ONT) long-read sequencing was performed at CRG, integrating the CapTrap cDNA library preparation method (8) with the CLS approach to produce over 1.5 billion raw reads, which were processed using the CRG LyRic pipeline (https://github.com/guigolab/LyRic) to generate a collection of full-length transcript models for potential integration into GENCODE annotation (see Data Access). This required the development of an annotation workflow that could create thousands of transcript models at a level of quality that approached that of manual annotation. Thus, we created TAGENE, a manually supervised annotation pipeline that was fine-tuned via extensive iterative testing. In this way, over 140 000 and 132 000 novel lncRNA transcripts were added to human and mouse— ∼3- and 6-fold increases in these respective counts—with the vast bulk first appearing in versions v47 for human and M36 for mouse (Table 1). TAGENE is now being further developed to facilitate the annotation of long-read data within protein-coding genes and will be redeployed on additional long-read datasets as they emerge.

In parallel to our in-house work, GENCODE is also helping the wider community coalesce to find the best methods for long-read alignment, quantification and quality control. Our project—particularly the UCSC, CRG and EMBL-EBI groups—played a significant role in the recently completed Long-Read RNAseq Genome Annotation Assessment Project (LRGASP) (9), an international ‘bake-off’ challenge whereby independent groups tested their methods for processing standardized sets of transcriptomics data. We aided in the project’s design, the generation of sequencing data, and experimental validation and contributed to the supporting infrastructure. Furthermore, the ‘ground truth’ annotations, which served as the benchmark for evaluating all submissions in specific challenges, were provided by expert annotators at EMBL-EBI.

### Annotating coding sequences

Our ultimate goal in protein annotation is to classify all bona fide CDSs in human and mouse. It can be assumed that GENCODE is missing CDS in both species and also includes CDS annotations that will subsequently be judged as false. Traditionally, our project has appraised potential CDS according to experimental data and evolutionary arguments, and the prospects for both methods continue to advance. Within GENCODE, the CNIO evaluates mass spectrometry support for existing proteins and prospective new annotations for both human and mouse. We are also now working closely with HUPO-HPP (10) and PeptideAtlas (11) in the characterization of non-canonical translations, as discussed below. Meanwhile, GENCODE evolutionary analyses are centered around the resources produced at MIT, especially in deploying the PhyloCSF algorithm (12,13). We emphasize that—in our experience—these analytical strands are best employed in conjunction and considered in collaboration.

The drive towards complete CDS annotation happens at two levels. Firstly, we aim to identify all protein-coding *genes* in both species; secondly, we aim to classify all genuine protein *isoforms* within these loci. The latter remains a trickier proposition. For example, while proteomics data can confirm that a given locus is protein-coding, the supporting peptides might not distinguish putative isoforms. Meanwhile, evolutionary analysis can provide strong support for the coding potential of a given isoform. However, the rate and extent to which additional isoforms arise as evolutionary novelties remains unclear. For these reasons, we have to date pursued a permissive annotation policy regarding isoforms, whereby alternative transcripts will generally be annotated as coding where the translation appears to be mechanistically plausible. Ultimately, we are often essentially certain that a given gene is protein-coding, but substantially less confident in assessing the true complement of isoforms.

Thus, progress here is more easily measured by tracking changes in gene-level annotation. Over the last 20 years, the number of human protein-coding genes annotated by GENCODE has gradually reduced, with 19 433 in v47. This largely reflects the removal or recharacterization of protein-coding genes that first appeared as *ab initio* predictions during the earliest years of genome annotation and which were subsequently reappraised as containing no merits as such based on experimental data or evolutionary analysis. For human, our major drive in this regard was carried out several years ago (6), and in fact the count of protein-coding genes now shows a modest increase over the last few releases; this results from targeted efforts which are discussed below. The situation in mouse is similar, although here the drive to remove bogus protein-coding genes was more recently instigated, and this explains the net fall of 198 protein-coding genes between releases M30 and M36 (Table 1).

Today GENCODE continues to work closely with RefSeq (14), UniProtKB (15) and the HUGO Gene Nomenclature Committee (HGNC) (16) as part of a shared annotation ‘ecosystem,’ whereby we are actively aiming to standardize our protein catalogs, leading with human efforts. Thus, discordant entries come forward for discussion during projects such as MANE (discussed below) and GIFTS (a ‘behind the scenes’ drive aiming to harmonize GENCODE annotations with UniProt proteins).

### Protein coding versus pseudogenes

**Figure 1.**
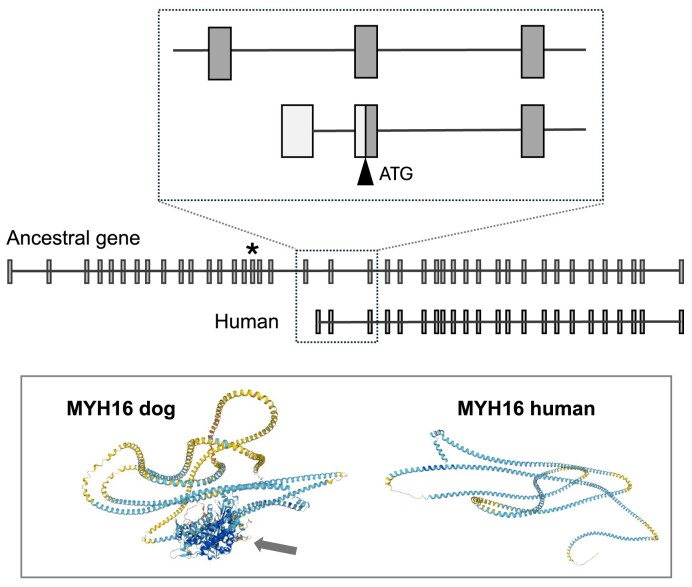
An example of a new protein-coding gene annotation was added from a former pseudogene annotation. Myosin heavy chain 16 (*MYH16*) is a former pseudogene that has now been annotated as protein-coding in GENCODE. The ancestral copy of the locus (center image, top model) has 43 exons, all of which are present in the human genome. However, exon 16 contains a fixed 2bp deletion at chr7:99 265 138–99 265 139 on GRCh38 (asterisk), which introduces a frameshift into the CDS and would thus be anticipated to be a loss of function mutation. Transcriptomics data (not shown) indicate that the human locus is nonetheless transcribed almost exclusively via an alternative first exon within intron 19–20 (lower model in upper inset image). This transcript is predicted to have a CDS based on an ‘internal’ ATG initiation codon found within the ancestral CDS (light shading for UTR sequence; dark for CDS), giving an 1143aa translation. This translation product—highly similar to UniProt human entry Q9H6N6—can be seen to have entirely lost the myosin head domain in the predicted Alphafold structure, leaving only the myosin tail (lower panel). The ancestral protein structure is illustrated by the dog UniProt entry F1PT61, with the head domain arrowed. Nonetheless, the human translation is well supported by proteomics data, with 36 unique mapping peptides corresponding to the UniProt entry in PeptideAtlas build 2024–01 (not shown). As such, GENCODE now annotates this locus as protein-coding. Its functionality remains obscure, however.

We can identify two modes by which protein-coding genes are added to GENCODE. First, certain loci are previously annotated as pseudogenes before new evidence shifts the balance of probability toward them being protein coding. Figure 1 represents the intriguing case of myosin heavy chain 16 (*MYH16*), a long-time human pseudogene recently adjudged as protein coding. The loss of function of this muscle protein via a disabling mutation was previously identified as a key step in reducing jaw size in our species compared to other apes (17). However, modern datasets indicate that *MYH16* is transcribed and translated from a modulated CDS significantly truncated at the 5′ end. While the functionality of this protein remains obscure, we consider the locus to be most likely protein coding, noting that we make decisions in this regard not as an appeal to certainty rather as to how we assess the balance of probability.

In other cases, GENCODE has switched protein-coding genes to pseudogenes, as the coding status of the locus is reappraised as being against the balance of probability. Such decisions are made in collaboration with our allied reference annotation projects. While removing false proteins is of clear practical value, annotating pseudogenes is also important when understanding organismal biology. Thus, annotating human and mouse pseudogenes remains a core GENCODE activity led by computational work at Yale University. A present drive is the characterization of pseudogenes in the genomes of mouse laboratory strains (18). It is becoming clear that pseudogenes are an excellent marker for genome changes at the structural level, exhibiting—for example—high dynamicity within complex regions, including segmental duplications. This has clear implications for pangenome annotation, as will be discussed below. Our work also indicates that pseudogenes also have the potential to act as functional elements driven by their transcription. Taking advantage of the large-scale RNA-seq datasets from PsychENCODE (19), we are examining the possibility that differentially expressed pseudogenes may be relevant to psychiatric disorders.

In practice, there are many loci where it is uncertain whether they are protein-coding or pseudogenic. For example, the enzyme ureidoimidazoline (2-oxo-4-hydroxy-4-carboxy-5-) decarboxylase (*URAD*) plays a key role in uric acid degradation in mammals. Nonetheless, this metabolic pathway is known to be completely absent in human (20) and there is no evidence for the existence of the protein. However, the CDS of the URAD gene is fully intact in our genome, and the locus is transcribed. Thus, *URAD* remains protein-coding in GENCODE, according to the possibility that the locus imparts some unknown, modulated function. This position could change in the future, although we recognize the philosophical difficulty in proving that a gene has no function.

### Annotating non-canonical translation

Other new protein-coding genes were previously unknown in any sense. We have had great success identifying missing proteins using PhyloCSF (13), which, in effect, scores the protein-coding potential of a DNA sequence according to the likelihood that it has evolved as coding versus non-coding. Today, there are unlikely to be any large proteins remaining to be directly identified by this method in human or mouse. However, the discovery of ‘microproteins’ (CDS under 100aa) is potentially a different story. This topic is of rapidly growing interest in the genomics community (21), and is a major facet of the drive to characterize non-canonical (i.e. unannotated) translation more broadly. The need for progress in annotation is demanded by the increasing usage of Ribo-seq, where a given assay typically finds thousands of translated open reading frames (ORFs) not annotated by GENCODE. We have now produced an initial consensus catalogue of 7264 human Ribo-seq ORFs using a collaborative community model (22), and these efforts are continuing in new phases.

PhyloCSF analysis indicates that very few Ribo-seq ORFs are under selective pressure as CDS within the mammalian order, and indeed, the majority are conserved only between higher primate lineages (23). While making this catalog, we annotated just 10 Ribo-seq ORFs as new protein-coding genes. At the same time, we find low support for the protein-coding potential of Ribo-seq ORFs based on proteomics data from whole-cell tryptic digests. However, working with the HUPO-HPP project, PeptideAtlas and other experts in the field, we are now examining the coding potential of Ribo-seq ORFs based on immunopeptidomics data; peptides that are naturally presented on the cell surface by the major histocompatability complex following cellular digestion *in situ* (24). We are helping to develop a community-standard methodology for using such data in reference annotation and find that over 1000 translations present preliminary evidence of support (manuscript under review). Nonetheless, the biological interpretation of these findings is not straightforward (25), as the data demonstrate protein existence but not actual function. Thus, GENCODE does not currently annotate Ribo-seq ORFs as novel protein-coding genes when the only support comes from immunopeptidomics data.

It could, therefore, be that certain Ribo-seq ORFs have no real physiological importance. Alternatively, it is now clear that translation has alternative modes of function that must be appraised through other methods. In particular, there is now a well-developed understanding of the role of upstream ORFs (uORFs) in the control of protein-coding gene expression (26), and GENCODE is building a new infrastructure for such annotations. To accompany this work, building on observations that many Ribo-seq ORFs are deeply conserved and yet lack PhyloCSF or proteomics evidence to support their protein-coding nature (22), we are now developing an algorithm to measure evolutionary constraint on an ORF independent of constraint on its encoded amino acid sequence.

One approach to distinguishing regulatory short ORFs from ones that function at the protein level is by comparing the predicted biophysical properties of their hypothetical translations to those of known short proteins. Still, past studies of such properties have typically used long lists of candidate proteins that include many false positives. To address this, we generated a ‘gold standard’ list of 173 proteins of no >70 amino acids with high confidence of function at the protein level based on evolutionary or experimental evidence, and reported statistics on their biophysical properties (27). This could help in the identification and annotation of putative microproteins, especially those for which experimental evidence currently proves elusive.

### The extended gene

In clinical science, variant interpretation pathways are still primarily centered around CDS annotated by GENCODE and RefSeq. However, non-coding regions of the genome are also highly important in function and relevance to disease, and clinical workflows are now taking them into account (28). GENCODE has always placed a strong focus on the annotation of the untranslated regions (UTRs) of protein-coding gene transcripts, which can be complex due to both alternative splicing and the usage of variable transcript start sites (TSSs) and polyadenylation (pA) sites. We are now working to improve UTR annotation further. First, many models are incomplete at their 5′ and 3′ ends; they do not contain true TSS and/or pA sites and so also lack accurate UTRs. This is problematic along several lines. When a transcript structure is incomplete, it can be difficult to judge the correct biotype of the model, especially if it contains a valid CDS. Similarly, our work on cataloguing uORFs as part of the study of non-canonical translation is undermined by incomplete 5′ UTR annotations. Also, the truncation of 3′ UTRs can have specific consequences for RNAseq quantification based on poly-A priming, whereby entire genes can incorrectly show up as non-expressed (29).

The MANE Collaboration (see below) has led to improvement here, as almost all MANE Select transcripts have accurately called TSS and pA sites as part of the process (30). Nonetheless, it is clear that the genome contains substantial amounts of UTR sequences that are not captured by MANE at present, and we are working to improve this situation. For 3′ UTRs, the study by Pool *et al.* led us to develop a workflow that leverages long-read datasets—especially from CLS—and pA-seq libraries to identify 3′ UTRs that can be extended in a semi-supervised computational manner; over a hundred extensions have been made during preliminary work. Similar efforts are being made at the 5′ ends, here using CAGE (31) and RAMPAGE (32) data to accompany the long-read libraries. Furthermore, we are also now deploying deep-learning models developed by the team at Stanford University, to assist annotation for the first time. We initially focused on improving TSS annotations using ProCapNet, a neural network that can accurately predict base-resolution initiation profiles from PRO-cap experiments using local DNA sequence (33). ProCapNet’s powerful interpretation framework reveals a comprehensive sequence motif lexicon of transcription initiation that includes known and novel variants of core promoter motifs and other specific TF motifs. It enables the identification of predictive motifs in actively transcribed regulatory elements, including enhancers, thereby guiding higher-resolution annotation of TSSs based on sequence elements.

Accurate TSSs can also anchor gene promoters, which are ubiquitous in controlling gene expression and are substantially important in phenotype and disease. Furthermore, promoters are the genomic sites contacted by long-distance enhancer elements, which are themselves also of major scientific and clinical interest. Until now, GENCODE has not annotated regulatory elements, and we consider that this may be one reason why downstream interpretative projects typically remain transcript-focused. Instead, projects such as ENCODE (34) and Ensembl Regulation (35) annotate promoters and enhancers in parallel, largely via the processing of massive datasets produced by genomic assays such as ChIP-seq and ATAC-seq. ‘Experiment-based’ annotations of this kind provide vital insights into the regulome, but our view is that the reach and utility of such resources would be expanded if they were tied directly into gene annotation. Our route to this is the new concept of the GENCODE ‘promoter window,’ and the first public catalog has now been finalized. Briefly, the promoter window for each protein-coding gene is defined and fixed as the 1000 bp immediately upstream of the MANE Select TSS. While promoter regions are of variable size in reality, this window was chosen as any promoter annotations for a given gene provided by Ensembl Regulation and ENCODE are highly likely to overlap with this sequence. Also, as noted, a ‘true’ TSS should in theory always colocalize with a promoter element. The GENCODE promoter thus provides a ‘window’ into the experimental data, and represents a new gene-centric gateway for the user to access this rich knowledge. These windows can also offer stability and standardization, as they are tied to the MANE Select transcript, which is unchanging.

The definition of promoters windows will then help us achieve the next goal of our work to catalog the ‘extended gene,’ which is to produce gene annotations integrated with their cell-type-specific regulatory circuitry. A necessary first step towards this goal is to comprehensively catalog enhancer–gene interactions (36). Several automated methods for enhancer–gene linking, including enhancer activity/gene expression correlations (37) and the activity-by-contact model (38,39), show promise for elucidating complex interactions. At present, MIT has made significant advancements on behalf of GENCODE by developing and utilizing resources such as EpiMap (37), which catalogs tissue and cell-type-specific enhancer–gene interactions, and by developing methods to integrate these enhancer–gene interactions with single-cell ATAC-seq.

### Developing GENCODE for the user

**Figure 2.**
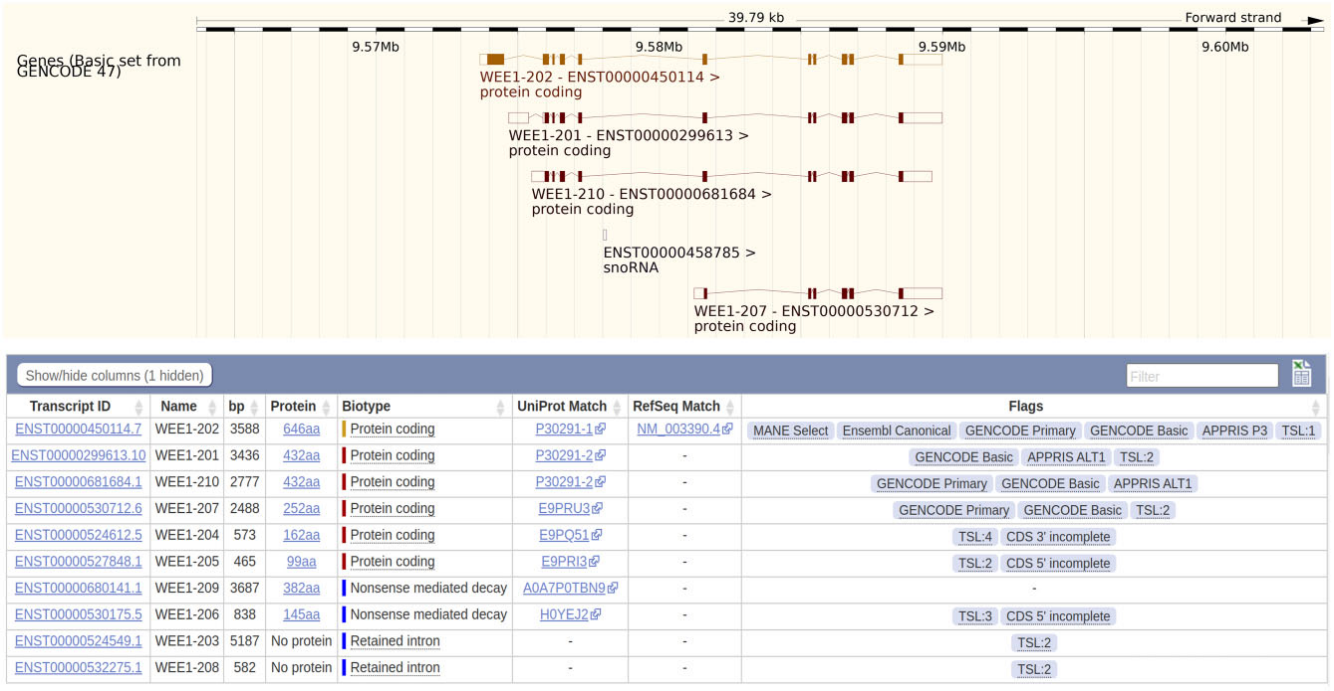
An Ensembl genome browser view of human protein-coding gene *WEE1*. Certain sections of the webpage have been omitted for clarity. Here, the Basic set is displayed within the annotation view (top), which thus shows only the four transcripts within the locus that have been annotated with full-length CDS. However, as the transcript table shows (bottom), *WEE1* contains ten transcript models, six of which are annotated as protein-coding. WEE1-202 is the MANE Select choice, with its RefSeq match displayed, and as such it is automatically considered as the single Ensembl Canonical model by the Ensembl project. WEE1-202 is also included in the GENCODE Primary set, as are additional models WEE1-210 and WEE1-207. In contrast, the fact that models WEE1-204 and WEE1-205 have partial CDS keeps them out of both the Basic set and GENCODE Primary, while the other four models (WEE1-203, WEE1-206, WEE1-208 and WEE1-209) are annotated as either nonsense mediated decay candidates or else models that contain retained introns (i.e. introns that have not been spliced out). Tags pertaining to APPRIS support are also visible (P for principal, ALT for alternative isoform; see https://apprisws.bioinfo.cnio.es/). Finally, the TSL tag refers to Transcript Support Level, which we consider in the process of being superseded by the functionality of Basic and GENCODE Primary. It highlights the level of support for a transcript model according to information from traditional mRNA and expressed sequence tag evidence sets.

Ultimately, the success of GENCODE is judged according to the scientific and clinical insights gained by those studies that use our resources. For this potential to be maximized, we are required to consider deep questions of usability. This is challenging; as the complexity of our resources increases, the natural tendency will be for usability to move in the opposite direction. The point is especially relevant to transcriptomics, where—according to our view of the data—it will soon become routine for protein-coding genes in human and mouse to contain tens or even hundreds of transcripts, many of uncertain functionality. Figure 2 presents an Ensembl genome browser view of the human *WEE1* gene, used here to illustrate certain solutions to this problem.

As noted, the improvement of CDS annotation represents ongoing work for GENCODE. Meanwhile, users typically, in practice, choose to work with a smaller number of transcripts per protein-coding gene, with a view to only including models of known or suspected functionality. Working with RefSeq, we released the first human MANE Select transcript set into the public domain in 2018 (30). These represent the choice jointly made by the two projects of the transcript for each protein-coding gene that is recommended for those situations where only a single transcript is needed. This choice was made according to a series of metrics, including transcriptomics support, evidence of evolutionary protein constraint via PhyloCSF, and knowledge of clinical relevance. At the time of release v47, all but a handful of protein-coding genes have a MANE Select transcript.

Nonetheless, a single transcript will often not capture the full suite of functionally important and potentially clinical relevant sequences in a given gene. Thus, MANE is expanding to include additional MANE Plus Clinical transcripts, which is necessary to allow the reporting of all known pathogenic or likely pathogenic clinical variants; 64 transcripts have been annotated so far. In the meantime, GENCODE is offering a new option for users who do not wish to include the full transcriptional complexity in their workflow but do want to try and maximize the inclusion of known or suspect functional elements. GENCODE Primary includes all MANE Select and MANE Plus Clinical Transcripts, together with a minimal set of additional transcripts that have been computationally identified as having features of interest not represented by MANE. In particular, this includes additional exons and splice junctions that are highly expressed as judged by the recount3 resource (a massive, standardized reprocessing of publicly available RNAseq datasets) (40) and CDS regions with conservation evidence from PhastCons (41) or protein constraint evidence from PhyloCSF (13). GENCODE Primary has been made initially available for human (v47), with mouse to follow, and it at present considers only full-length protein-coding transcripts for inclusion. This first human release contains an average of two transcripts per protein-coding gene and will now become the default annotation view in the UCSC and Ensembl genome browsers. An alternative solution is offered by the GENCODE Basic set, which includes all models annotated as protein-coding that are considered as full-length, i.e. with a complete CDS. Finally, the CNIO continues to develop its APPRIS pipeline (42), which advises users on transcript and protein isoform prioritization for human and mouse (as well as other species) based on functional scores predicted by the TRIFID algorithm (43). The APPRIS ‘principal isoforms’ set for human protein-coding genes show high concordance with the MANE Select catalogue (44). As illustrated in Figure 2, MANE, Primary, Basic and APPRIS information is included in the Ensembl Transcript table for *WEE1* as for every gene, and these tags are also present in the annotation files offered for download.

### Towards pangenome annotation

GENCODE human annotation efforts remain focused on the reference genome GRCh38. However, for each release we also provide ‘liftover’ annotations to the previous GRCh37/hg19 assembly, produced computationally at UCSC. Note, however, that MANE transcripts are only available on GRC38. Meanwhile, for mouse, we have now completed the move of our annotations from GRCm38 to GRCm39. In the last couple of years, our focus has started to shift to other genomes of these species. For human, we now have full availability of the gap-free CHM13 genome produced by the T2T consortium (45). Moreover, the T2T genome is now one of many high-quality human genomes produced based on modern sequencing protocols; in particular, the Human Pangenome Reference Consortium (HPRC) has now released the first draft pangenome assembly, which is based on 47 phased, diploid assemblies from a cohort of genetically diverse individuals (46). The number of assemblies in the human pangenome is expected to grow substantially. For mouse, high-quality genomes are now becoming available; mouse has its own T2T project nearing completion, while the Mouse Genomes Project has produced numerous high-quality builds for various laboratory strains (47).

We are now making substantial efforts to understand how to produce reference annotations for such genomes. It is not practical to manually annotate additional genomes like the human and mouse reference assemblies were annotated. Neither is it necessary; initial efforts by GENCODE using the Comparative Annotation Toolkit (CAT) developed at UCSC (48) and Ensembl liftover tools indicate that computational mapping of annotations between same-species genomes is broadly effective. The major questions concern what to do in regions that are not amenable to computational annotation, which include—for example—segmental duplications that exhibit copy number variation, as well as regions that were not present on the reference assembly, such as the p-arms of human acrocentric chromosomes. Preliminary manual annotation efforts by GENCODE in collaboration with the HPRC are helping to illuminate the problem. For example, our first analysis of the T2T assembly revealed that this genome contains the actual protein-coding gene for *WASHC1*, and it now appears that the various paralogs found on the GRCh38 assembly are potentially pseudogenic (49). There is also the question of which transcriptomics datasets to use for pangenome annotations. Here, CRG is starting to work on the generation of full-length transcripts from a diversity of genetic backgrounds linked to HPRC.

## Conclusion

In 2025 GENCODE may be considered as a mature gene annotation project. Nonetheless, we expect it will remain a work in progress for years to come. New technologies and methodologies have the potential to aid the annotation process greatly, but their application also presents challenges to our project. In particular, it is now clear that both the human and mouse genomes express far more transcripts than are currently annotated, while the substantial majority that have been or can be annotated remain obscure regarding their functionality. We see the route to progress via an integrated approach, whereby any useful, high-quality data from disparate sources can be brought together into a unified annotation workflow. Furthermore, it is now evident that new computational methods based on machine learning will have a crucial role to play, and we have already taken the first steps in this regard. However, while GENCODE remains a highly dynamic project under the surface, our users naturally favor stability and—in general—simplicity. Thus, the current drive for GENCODE is not just to capture genomic and cellular complexity *in silico* but also to produce a resource that parses the most relevant information in a manner that is easily understood and accessible.

## Data Availability

A new GENCODE release is produced up to four times each year for both human and mouse. Each release is made freely available immediately upon release from the Ensembl website (https://www.ensembl.org) and the GENCODE webportal (https//www.gencodegenes.org), with a release on the UCSC Genome Browser shortly after that (https://genome.ucsc.edu/). GENCODE is currently the default annotation in both genome browsers, and is embedded in numerous genomics and clinical projects. The current human release is GENCODE 47, and the current mouse release is GENCODE M36 (October 2024). Additional information and previous releases can be found at https//www.gencodegenes.org. MANE annotations are available from the Ensembl and RefSeq NCBI websites and can be viewed on both the Ensembl and UCSC genome browsers. To expedite public access to updated annotation between releases, all annotation changes are made freely available within 24 h via the ‘GENCODE Annotation Updates’ Track Hub, accessed at both the Ensembl and UCSC genome browsers. GENCODE has been designated a Global Core Biodata Resource by the Global Biodata Coalition. GENCODE produces the human and mouse gene annotation for the Ensembl project, in collaboration with Ensembl. Human 47 and mouse M36 are contained within Ensembl release e113. Programmatic access to the GENCODE gene sets is possible via the extensive Ensembl Perl API and the language-agnostic Ensembl REST API (50). Programmatic access facilitates advanced genome-wide analysis such as retrieval of supporting features and associated gene trees. Examples of REST endpoint usage and starter scripts in different languages are at https://rest.ensembl.org. Other interfaces include the Ensembl FTP site (ftp://ftp.ensembl.org/pub/), which includes gene sets in GFF3, Genbank and GTF formats and full download of the complete Ensembl databases. GENCODE-specific training materials and GENCODE-focused workshops from the Ensembl Outreach team are available via the Ensembl Training portal (http://training.ensembl.org) and EMBL-EBI (https://www.ebi.ac.uk/training/on-demand), and are regularly presented at online and in-person training events. Further information on the results of the GENCODE CLS pipeline to produce a collection of full-length high-quality transcripts—including access to the human and mouse master tables of transcript models prior to full annotation—is available here: https://github.com/guigolab/gencode-cls-master-table. All raw transcriptomics data produced by GENCODE to support the CLS work have been uploaded to the ENCODE data repository (see https://www.encodeproject.org/about/data-access/) and will be made publicly available as part of a manuscript describing this work, currently in preparation. Our resources are freely available at our web portal, www.gencodegenes.org, and via the Ensembl (https://www.ensembl.org) and UCSC genome browsers (https://genome.ucsc.edu/).

## References

[1] Harrow J. , Denoeud F., Frankish A., Reymond A., Chen C.-K., Chrast J., Lagarde J., Gilbert J.G.R., Storey R., Swarbreck D. et al. GENCODE: producing a reference annotation for ENCODE. Genome Biol. 2006; 7:S4.1–S4.9. 10.1186/gb-2006-7-s1-s4 PMC1810553 16925838

[2] Lander E.S. , Linton L.M., Birren B., Nusbaum C., Zody M.C., Baldwin J., Devon K., Dewar K., Doyle M., FitzHugh W. et al. Initial sequencing and analysis of the human genome. Nature. 2001; 409:860–921. 11237011 10.1038/35057062

[3] Guigó R. , Flicek P., Abril J.F., Reymond A., Lagarde J., Denoeud F., Antonarakis S., Ashburner M., Bajic V.B., Birney E. et al. EGASP: the human ENCODE genome annotation Assessment Project. Genome Biol. 2006; 7:S2.1–S2.31. 10.1186/gb-2006-7-s1-s2 PMC1810551 16925836

[4] Benson D.A. , Cavanaugh M., Clark K., Karsch-Mizrachi I., Lipman D.J., Ostell J., Sayers E.W. GenBank. Nucleic Acids Res. 2013; 41:D36–D42. 23193287 10.1093/nar/gks1195 PMC3531190

[5] Ingolia N.T. , Ghaemmaghami S., Newman J.R.S., Weissman J.S. Genome-wide analysis in vivo of translation with nucleotide resolution using ribosome profiling. Science. 2009; 324:218–223. 19213877 10.1126/science.1168978 PMC2746483

[6] Frankish A. , Carbonell-Sala S., Diekhans M., Jungreis I., Loveland J.E., Mudge J.M., Sisu C., Wright J.C., Arnan C., Barnes I. et al. GENCODE: reference annotation for the human and mouse genomes in 2023. Nucleic Acids Res. 2023; 51:D942–D949. 36420896 10.1093/nar/gkac1071 PMC9825462

[7] Lagarde J. , Uszczynska-Ratajczak B., Carbonell S., Pérez-Lluch S., Abad A., Davis C., Gingeras T.R., Frankish A., Harrow J., Guigo R. et al. High-throughput annotation of full-length long noncoding RNAs with capture long-read sequencing. Nat. Genet. 2017; 49:1731–1740. 29106417 10.1038/ng.3988 PMC5709232

[8] Carbonell-Sala S. , Perteghella T., Lagarde J., Nishiyori H., Palumbo E., Arnan C., Takahashi H., Carninci P., Uszczynska-Ratajczak B., Guigó R. CapTrap-seq: a platform-agnostic and quantitative approach for high-fidelity full-length RNA sequencing. Nat. Commun. 2024; 15:5278. 38937428 10.1038/s41467-024-49523-3 PMC11211341

[9] Pardo-Palacios F.J. , Wang D., Reese F., Diekhans M., Carbonell-Sala S., Williams B., Loveland J.E., De María M., Adams M.S., Balderrama-Gutierrez G. et al. Systematic assessment of long-read RNA-seq methods for transcript identification and quantification. Nat. Methods. 2024; 21:1349–1363. 38849569 10.1038/s41592-024-02298-3 PMC11543605

[10] Omenn G.S. , Lane L., Overall C.M., Lindskog C., Pineau C., Packer N.H., Cristea I.M., Weintraub S.T., Orchard S., Roehrl M.H.A. et al. The 2023 report on the Proteome from the HUPO Human Proteome Project. J. Proteome Res. 2024; 23:532–549. 38232391 10.1021/acs.jproteome.3c00591 PMC11026053

[11] Desiere F. , Deutsch E.W., King N.L., Nesvizhskii A.I., Mallick P., Eng J., Chen S., Eddes J., Loevenich S.N., Aebersold R. The PeptideAtlas project. Nucleic Acids Res. 2006; 34:D655–D658. 16381952 10.1093/nar/gkj040 PMC1347403

[12] Lin M.F. , Jungreis I., Kellis M. PhyloCSF: a comparative genomics method to distinguish protein coding and non-coding regions. Bioinformatics. 2011; 27:i275–i82. 21685081 10.1093/bioinformatics/btr209 PMC3117341

[13] Mudge J.M. , Jungreis I., Hunt T., Gonzalez J.M., Wright J.C., Kay M., Davidson C., Fitzgerald S., Seal R., Tweedie S. et al. Discovery of high-confidence human protein-coding genes and exons by whole-genome PhyloCSF helps elucidate 118 GWAS loci. Genome Res. 2019; 29:2073–2087. 31537640 10.1101/gr.246462.118 PMC6886504

[14] O’Leary N.A. , Wright M.W., Brister J.R., Ciufo S., Haddad D., McVeigh R., Rajput B., Robbertse B., Smith-White B., Ako-Adjei D. et al. Reference sequence (RefSeq) database at NCBI: current status, taxonomic expansion, and functional annotation. Nucleic. Acids. Res. 2016; 44:D733–D745. 26553804 10.1093/nar/gkv1189 PMC4702849

[15] Consortium U.P. UniProt: the Universal Protein knowledgebase in 2023. Nucleic Acids Res. 2023; 51:D523–D531. 36408920 10.1093/nar/gkac1052 PMC9825514

[16] Seal R.L. , Braschi B., Gray K., Jones T.E.M., Tweedie S., Haim-Vilmovsky L., Bruford E.A. Genenames.Org: the HGNC resources in 2023. Nucleic Acids Res. 2023; 51:D1003–D1009. 36243972 10.1093/nar/gkac888 PMC9825485

[17] Stedman H.H. , Kozyak B.W., Nelson A., Thesier D.M., Su L.T., Low D.W., Bridges C.R., Shrager J.B., Minugh-Purvis N., Mitchell M.A. Myosin gene mutation correlates with anatomical changes in the human lineage. Nature. 2004; 428:415–418. 15042088 10.1038/nature02358

[18] Sisu C. , Muir P., Frankish A., Fiddes I., Diekhans M., Thybert D., Odom D.T., Flicek P., Keane T.M., Hubbard T. et al. Transcriptional activity and strain-specific history of mouse pseudogenes. Nat. Commun. 2020; 11:3695. 32728065 10.1038/s41467-020-17157-w PMC7392758

[19] PsychENCODE Consortium Akbarian S. , Liu C., Knowles J.A., Vaccarino F.M., Farnham P.J., Crawford G.E., Jaffe A.E., Pinto D., Dracheva S. et al. The PsychENCODE project. Nat. Neurosci. 2015; 18:1707–1712. 26605881 10.1038/nn.4156 PMC4675669

[20] Oda M. , Satta Y., Takenaka O., Takahata N. Loss of urate oxidase activity in hominoids and its evolutionary implications. Mol. Biol. Evol. 2002; 19:640–653. 11961098 10.1093/oxfordjournals.molbev.a004123

[21] Mohsen J.J. , Martel A.A., Slavoff S.A. Microproteins-discovery, structure, and function. Proteomics. 2023; 23:e2100211. 37603371 10.1002/pmic.202100211 PMC10841188

[22] Mudge J.M. , Ruiz-Orera J., Prensner J.R., Brunet M.A., Calvet F., Jungreis I., Gonzalez J.M., Magrane M., Martinez T.F., Schulz J.F. et al. Standardized annotation of translated open reading frames. Nat. Biotechnol. 2022; 40:994–999. 35831657 10.1038/s41587-022-01369-0 PMC9757701

[23] Sandmann C.-L. , Schulz J.F., Ruiz-Orera J., Kirchner M., Ziehm M., Adami E., Marczenke M., Christ A., Liebe N., Greiner J. et al. Evolutionary origins and interactomes of human, young microproteins and small peptides translated from short open reading frames. Mol. Cell. 2023; 83:994–1011. 36806354 10.1016/j.molcel.2023.01.023 PMC10032668

[24] Shapiro I.E. , Bassani-Sternberg M. The impact of immunopeptidomics: from basic research to clinical implementation. Semin. Immunol. 2023; 66:101727. 36764021 10.1016/j.smim.2023.101727

[25] Prensner J.R. , Abelin J.G., Kok L.W., Clauser K.R., Mudge J.M., Ruiz-Orera J., Bassani-Sternberg M., Moritz R.L., Deutsch E.W., van Heesch S. What can ribo-seq, immunopeptidomics, and proteomics tell us about the noncanonical proteome?. Mol. Cell. Proteomics. 2023; 22:100631. 37572790 10.1016/j.mcpro.2023.100631 PMC10506109

[26] Dever T.E. , Ivanov I.P., Hinnebusch A.G. Translational regulation by uORFs and start codon selection stringency. Genes Dev. 2023; 37:474–489. 37433636 10.1101/gad.350752.123 PMC10393191

[27] Whited A.M. , Jungreis I., Allen J., Cleveland C.L., Mudge J.M., Kellis M., Rinn J.L., Hough L.E. Biophysical characterization of high-confidence, small human proteins. Biophys. Rep. (NY). 2024; 4:100167. 10.1016/j.bpr.2024.100167 PMC11305224 38909903

[28] Ellingford J.M. , Ahn J.W., Bagnall R.D., Baralle D., Barton S., Campbell C., Downes K., Ellard S., Duff-Farrier C., FitzPatrick D.R. et al. Recommendations for clinical interpretation of variants found in non-coding regions of the genome. Genome Med. 2022; 14:73. 35850704 10.1186/s13073-022-01073-3 PMC9295495

[29] Pool A.-H. , Poldsam H., Chen S., Thomson M., Oka Y. Recovery of missing single-cell RNA-sequencing data with optimized transcriptomic references. Nat. Methods. 2023; 20:1506–1515. 37697162 10.1038/s41592-023-02003-w

[30] Morales J. , Pujar S., Loveland J.E., Astashyn A., Bennett R., Berry A., Cox E., Davidson C., Ermolaeva O., Farrell C.M. et al. A joint NCBI and EMBL-EBI transcript set for clinical genomics and research. Nature. 2022; 604:310–315. 35388217 10.1038/s41586-022-04558-8 PMC9007741

[31] Shiraki T. , Kondo S., Katayama S., Waki K., Kasukawa T., Kawaji H., Kodzius R., Watahiki A., Nakamura M., Arakawa T. et al. Cap analysis gene expression for high-throughput analysis of transcriptional starting point and identification of promoter usage. Proc. Natl Acad. Sci. U.S.A. 2003; 100:15776–15781. 14663149 10.1073/pnas.2136655100 PMC307644

[32] Batut P. , Gingeras T.R. RAMPAGE: promoter activity profiling by paired-end sequencing of 5’-complete cDNAs. Curr. Protoc. Mol. Biol. 2013; 104:Unit 25B.11. 24510412 10.1002/0471142727.mb25b11s104 PMC4372803

[33] Cochran K. , Yin M., Mantripragada A., Schreiber J., Marinov G.K., Kundaje A. Dissecting the cis-regulatory syntax of transcription initiation with deep learning. 2024; biorxiv doi:01 June 2024, preprint: not peer reviewed 10.1101/2024.05.28.596138.

[34] Project Consortium E.N.C.O.D.E. An integrated encyclopedia of DNA elements in the human genome. Nature. 2012; 489:57–74. 22955616 10.1038/nature11247 PMC3439153

[35] Harrison P.W. , Amode M.R., Austine-Orimoloye O., Azov A.G., Barba M., Barnes I., Becker A., Bennett R., Berry A., Bhai J. et al. Ensembl 2024. Nucleic Acids Res. 2024; 52:D891–D899. 37953337 10.1093/nar/gkad1049 PMC10767893

[36] Gschwind A.R. , Mualim K.S., Karbalayghareh A., Sheth M.U., Dey K.K., Jagoda E., Nurtdinov R.N., Xi W., Tan A.S., Jones H. et al. An encyclopedia of enhancer-gene regulatory interactions in the human genome. 2023; bioRxiv doi:13 November 2023, preprint: not peer reviewed 10.1101/2023.11.09.563812. PMC13471189 42457959

[37] Boix C.A. , James B.T., Park Y.P., Meuleman W., Kellis M. Regulatory genomic circuitry of human disease loci by integrative epigenomics. Nature. 2021; 590:300–307. 33536621 10.1038/s41586-020-03145-z PMC7875769

[38] Fulco C.P. , Nasser J., Jones T.R., Munson G., Bergman D.T., Subramanian V., Grossman S.R., Anyoha R., Doughty B.R., Patwardhan T.A. et al. Activity-by-contact model of enhancer–promoter regulation from thousands of CRISPR perturbations. Nat. Genet. 2019; 51:1664–1669. 31784727 10.1038/s41588-019-0538-0 PMC6886585

[39] Hecker D. , Behjati Ardakani F., Karollus A., Gagneur J., Schulz M.H. The adapted Activity-by-Contact model for enhancer-gene assignment and its application to single-cell data. Bioinformatics. 2023; 39:btad062. 36708003 10.1093/bioinformatics/btad062 PMC9931646

[40] Wilks C. , Zheng S.C., Chen F.Y., Charles R., Solomon B., Ling J.P., Imada E.L., Zhang D., Joseph L., Leek J.T. et al. recount3: summaries and queries for large-scale RNA-seq expression and splicing. Genome Biol. 2021; 22:323. 34844637 10.1186/s13059-021-02533-6 PMC8628444

[41] Siepel A. , Bejerano G., Pedersen J.S., Hinrichs A.S., Hou M., Rosenbloom K., Clawson H., Spieth J., Hillier L.W., Richards S. et al. Evolutionarily conserved elements in vertebrate, insect, worm, and yeast genomes. Genome Res. 2005; 15:1034–1050. 16024819 10.1101/gr.3715005 PMC1182216

[42] Rodriguez J.M. , Pozo F., Cerdán-Vélez D., Di Domenico T., Vázquez J., Tress M.L. APPRIS: selecting functionally important isoforms. Nucleic Acids Res. 2022; 50:D54–D59. 34755885 10.1093/nar/gkab1058 PMC8728124

[43] Pozo F. , Martinez-Gomez L., Walsh T.A., Rodriguez J.M., Di Domenico T., Abascal F., Vazquez J., Tress M.L. Assessing the functional relevance of splice isoforms. NAR Genom. Bioinform. 2021; 3:lqab044. 34046593 10.1093/nargab/lqab044 PMC8140736

[44] Pozo F. , Rodriguez J.M., Vázquez J., Tress M.L. Clinical variant interpretation and biologically relevant reference transcripts. NPJ Genom. Med. 2022; 7:59. 36257961 10.1038/s41525-022-00329-6 PMC9579139

[45] Nurk S. , Koren S., Rhie A., Rautiainen M., Bzikadze A.V., Mikheenko A., Vollger M.R., Altemose N., Uralsky L., Gershman A. et al. The complete sequence of a human genome. Science. 2022; 376:44–53. 35357919 10.1126/science.abj6987 PMC9186530

[46] Liao W.-W. , Asri M., Ebler J., Doerr D., Haukness M., Hickey G., Lu S., Lucas J.K., Monlong J., Abel H.J. et al. A draft human pangenome reference. Nature. 2023; 617:312–324. 37165242 10.1038/s41586-023-05896-x PMC10172123

[47] Lilue J. , Doran A.G., Fiddes I.T., Abrudan M., Armstrong J., Bennett R., Chow W., Collins J., Collins S., Czechanski A. et al. Sixteen diverse laboratory mouse reference genomes define strain-specific haplotypes and novel functional loci. Nat. Genet. 2018; 50:1574–1583. 30275530 10.1038/s41588-018-0223-8 PMC6205630

[48] Fiddes I.T. , Armstrong J., Diekhans M., Nachtweide S., Kronenberg Z.N., Underwood J.G., Gordon D., Earl D., Keane T., Eichler E.E. et al. Comparative Annotation Toolkit (CAT)-simultaneous clade and personal genome annotation. Genome Res. 2018; 28:1029–1038. 29884752 10.1101/gr.233460.117 PMC6028123

[49] Cerdán-Vélez D. , Tress M.L. The T2T-CHM13 reference assembly uncovers essential WASH1 and GPRIN2 paralogues. Bioinform. Adv. 2024; 4:vbae029. 38464973 10.1093/bioadv/vbae029 PMC10924726

[50] Yates A. , Beal K., Keenan S., McLaren W., Pignatelli M., Ritchie G.R.S., Ruffier M., Taylor K., Vullo A., Flicek P. The Ensembl REST API: ensembl data for any language. Bioinformatics. 2015; 31:143–145. 25236461 10.1093/bioinformatics/btu613 PMC4271150

